# Prior task experience affects temporal prediction and estimation

**DOI:** 10.3389/fpsyg.2015.00916

**Published:** 2015-07-06

**Authors:** Simon Tobin, Simon Grondin

**Affiliations:** École de Psychologie, Université Laval, Québec, QC, Canada

**Keywords:** timing and time perception, task experience, expert performance, estimation, prediction, running

## Abstract

It has been shown that prior experience with a task improves temporal prediction, even when the amount of prior experience with the task is often limited. The present study targeted the role of *extensive* training on temporal prediction. Expert and intermediate runners had to predict the time of a 5 km running competition. Furthermore, after the race’s completion, participants had to estimate their running time so that it could be compared with the predicted time. Results show that expert runners were more accurate than intermediate runners for both predicting and estimating their running time. Furthermore, only expert runners had an estimation that was more accurate than their initial prediction. The results confirm the role of prior task experience in both temporal prediction and estimation.

## Introduction

Time perception, as opposed to other sensory modalities, does not rely on sensory receptors. As a consequence, researchers trying to explain time perception quickly turned into the direction of cognitive processes such as attention and memory (Roeckelein, 2008). While the role of attention in timing as been thoroughly discussed (see Brown, 2008, for a review), some aspects of the involvement of memory, especially long-term memory (LTM), are still understudied, as pointed out recently by many authors (Rattat and Droit-Volet, 2005b; Taatgen and van Rijn, 2011; Tobin and Grondin, 2012). Nonetheless, it should be noted that some aspects of LTM were studied in a timing research perspective, such as the lifespan of time intervals in memory (Gamache and Grondin, 2010), the interference between different temporal traces (Grondin, 2005) or between other task demands and memory traces (Ogden et al., 2008), the development of temporal memory (Rattat and Droit-Volet, 2005a,b, 2007), the effect of the number of presentations of a standard duration on temporal discrimination (Jones and Wearden, 2003; Grondin and McAuley, 2009; Grondin, 2012), the influence of pharmacological substances on temporal memory (Meck, 1983), and the EEG basis of memory traces (Ng et al., 2011).

Even if the involvement of LTM in timing did receive some attention lately, the actual corpus of knowledge in the literature is still thinner than one may wish. One particular overlooked aspect of LTM that has been recently brought up by Tobin and Grondin (2012) is the effect of prior experience with a task on the perceived duration of that task. Indeed, as many daily activities (for instance, driving to work) occurs routinely, it is very likely that one learns temporal information about recurring tasks, temporal information that in turn can improve temporal estimation. As a matter of fact, children as young as 4 years old can classify orderly activities like eating a cookie and watching a movie on the basis of their duration. This shows that children already have a representation of how long some tasks may last (Friedman, 1990).

One of the reason why the influence of prior experience with a task on timing has been overlooked until recently might simply be because it appears too obvious (Tobin and Grondin, 2012). It is logical to think that one uses experience about a task when such experience is available. Nonetheless, the influence of prior experience on timing clearly deserves empirical investigations for two main reasons. First, as many daily tasks happen more than once, many temporal judgments should occur in situations when prior experience with a task is available. Not taking prior experience with a task into account does not seem a very ecological way to address temporal perception, especially now that a growing number of researchers agree that time perception researches should turn to more ecological tasks (Tobin et al., 2010; Bisson et al., 2012; Matthews and Meck, 2014). Secondly, studying prior experience, as it was shown recently by Tobin and Grondin (2012), sheds light on the involvement of LTM in timing, an involvement that has long been overshadowed by the more prominent and studied role of attention.

### Prior Experience with a Task

The effect of prior experience with a task on timing may be explained by two main cognitive processes. First, as the task is repeated, its execution becomes automatized and requires less attention to perform, leaving more attentional resources for time monitoring. Since the amount of attention available for timing is strongly related to the accuracy of temporal judgments, it explains why the durations of trained tasks are more accurate than novel ones. This demonstration has been reported numerous times in the literature (see Block et al., 2010). The second aspect that could explain the effect of prior experience regards LTM. Indeed, through numerous repetitions of the task, one gains certain knowledge of how long the task lasts.

A recent study by Tobin and Grondin (2012) targeted the involvement of LTM by measuring how different levels of task duration knowledge affect temporal perception. They defined “task duration knowledge” as LTM stored knowledge about the duration of a task. Their study showed that task duration knowledge can improve temporal performance across different temporal tasks (verbal estimation and production) and duration range (from 30 to 90 s). Furthermore, this result was obtained by two distinct manipulations, both requiring the participation of elite athletes (swimmers). First, they compared the temporal perception of two automatized tasks, i.e., tasks with higher task duration knowledge than the other. Secondly, they altered the context in which a single task was performed in order to control the usage of task duration knowledge. In both cases, having more task duration knowledge, or performing the task in a context that allowed relying on task duration knowledge, enhanced the temporal judgments’ precision. In addition, they also performed a third experiment in which elite swimmers were asked to produce 36 s of visualization of a well known task (swimming) and another unknown task (climbing Mount Everest). This experiment further showed that the physical execution is not required to observe an effect of prior experience with a task as the temporal productions of the swimming task (familiar) were much more precise than that of the climbing one (unfamiliar).

While the task was not physically executed in this last experiment, it was still visualized. If no execution at all (whether physically or mentally) is performed, can prior experience with a task still enhance temporal perception? In other words, do elite athletes like those who participated in Tobin and Grondin (2012) simply know how long it takes them to cover certain distances? The best way to answer that question is to require a temporal prediction of participants with various expertise levels. Indeed, in the prediction task, the temporal judgment is made *before* the task is even executed, thus, the temporal judgment cannot rely on any cues related to the execution of the task but only on previous knowledge with the task at hand. Indeed, the attentional explanation of the effect of prior experience cannot apply to the prediction task; the temporal judgment can only rely on previously learned knowledge stored in LTM.

Thus, the first goal of the experiment is to extend the findings of the Tobin and Grondin (2012) study to the prediction task. In that regards, the literature already provides certain answers. Indeed, many experiments, although they did not use the terms task duration knowledge, did observe the effect of prior experience with a task on temporal prediction (Thomas et al., 2003, 2007; Thomas and Handley, 2008; see Halkjelsvik and Jørgensen, 2012, for a review).

For instance, Thomas et al. (2003) gave participants a little practice time (2 min) with the task before asking them a temporal prediction. It turned out that this simple 2 min of practice strongly increased the prediction performance. Furthermore, Roy et al. (2008) gave participants a single practice trial and further gave a temporal feedback about the duration of that trial for only half of the participants. When asked to make temporal prediction in the following trial, participants who received the temporal feedback were more accurate, showing that they used the information provided by the feedback to guide their next prediction. Finally, Roy and Christenfeld (2007, Experiment 2) compared the prediction of a task based on experience with the task. Indeed, participants had a practice block containing one, three or nine trials of the targeted task (origami). It turned out that the number of trials significantly affected temporal prediction. The number of practice trials affected the side of the error; participants with one practice trial overestimated the time it would take and participants with nine trials underestimated the time it would take.

The aforementioned studies suggest that prior experience with a task increases the precision of the temporal prediction, or changes the side of the error (from overestimation to underestimation). However, it should be noted that, in these experiments, the prior experience is often limited (from only a part of the task to nine repetitions of the task). Although their results were quite interesting, it appears necessary to study the effect of a more *extensive* prior experience. Indeed, as Tobin and Grondin (2012) pointed out in introducing the notion of task duration knowledge, this aspect of temporal perception is relevant for recurring tasks, tasks that are executed on a daily basis, again and again. Thus, although the previously cited experiments were well constructed and have a clear theoretical output, it does not show how the temporal prediction is affected by a level of prior experience that is *comparable* with other daily activities, like driving to work each day for many years.

As a result, one legitimate question arising is the following: what happens when one has an *extensive* training with the task, such as athletes do with their sport? Does the prediction reach an impressive accuracy level, as it is observed with temporal estimation (see Tobin and Grondin, 2012)? As far as we know, the only study that required the participation of experts (pianists) is the one reported by Boltz et al. (1998; Experiment 2). In their experiment, they compared the time prediction across novices and expert pianists for the execution of musical pieces varying in their degree of familiarity (i.e., identified as recently learned, well learned or extremely well learned). Their results show that for both experts and novices, the degree of familiarity had a significant effect on predicted time: the less familiar the melody was, the longer the predicted duration was. However, experts were surprisingly not better at predicting time than novices, which contradicts what may be expected on the basis of other previously cited studies (Thomas et al., 2003; Roy et al., 2008). Indeed, as prior task experience seems to increase time prediction accuracy, experts should have been better than novices. Two methodological aspects of their experiment may explain this non-significant result. First, participants were instructed to predict to the nearest 30 s. It might have leveled the predictions across the two groups and masked any significant difference that was within a 30-s margin. Furthermore, in music, the key temporal element might be the inter-note interval or tempo, not the overall duration. Hence, it might be best to study the effect of expertise on time prediction with a task in which the elapsed duration is fundamental, like in sports. This idea will be tested in the present experiment.

### Temporal Estimation

Using the prediction task opens the door to studying another relevant aspect of timing. Indeed, while a temporal prediction on its own is interesting, it is even more useful if it is compared with an assessment of the duration once the task is completed. Indeed, as the prediction cannot rely on active time monitoring, it is intriguing to assess how far the prediction is from the temporal estimation of the same task upon completion. Few studies compared directly temporal prediction and the subsequent estimation of the task once completed. Some studies did offer that comparison (Roy and Christenfeld, 2008), but used the retrospective paradigm. Such a paradigm means that participants were not told before the start of the duration to be timed that time estimation would be required. Hence, in a retrospective timing task, participants learn the time estimation requirement afterward. Though retrospective estimates are valuable measures of timing and deserve more empirical investigation (see Tobin et al., 2010; Bisson et al., 2012), it would probably be more relevant, when comparing temporal prediction and estimation, to use the prospective paradigm. In this paradigm, participants are told in advance that a temporal judgment will be required after completing the task. Hence participants can allow more attentional resources to time monitoring, explaining why time estimates in the prospective paradigm are most often reported as more precise than time estimates in retrospective conditions (Block and Zakay, 1997; Block et al., 2010). By using the prospective paradigm, this experiment should answer the following question: if one puts all its attentional resources into timing its running performance, can its estimation be more precise than the initial prediction or there is no gain to be expected?

The few studies left that compared time estimation (prospectively) and prediction do not allow for a clear picture of how these two judgment types differ. First, the Boltz et al.’s (1998) experiment showed that, for expert pianists, the estimated duration was more accurate than the predicted duration. However, the difference between temporal estimation and prediction of novices was mediated by the familiarity with the melody. Indeed, the estimations were more accurate than the predictions for only two of the three familiarity levels (novel and well trained). This improvement was not recorded for the extremely well trained melodies.

On the opposite, Burt and Kemp (1994) found large differences when comparing the prediction and estimation of daily activities (like buying stamps or sorting cards). Indeed, the temporal estimation accuracy after the task’s completion was steeply increased when compared to the actual prediction. Hence, the difference between temporal prediction and estimation appears unclear so far and might be mediated by the level of familiarity or prior experience with the task, as suggested by the results of Boltz et al. (1998).

### The Present Study

For the experiment’s purpose, expert and intermediate runners were recruited and had to predict how long it would take them to run a 5 km race. Participants were also required to estimate their completion time immediately after the finish line.

Since prior task experience seems to improve temporal judgments, it was expected the more experienced runners to have the best temporal prediction and estimation. Furthermore, we expected the temporal estimation to be more accurate than the initial time prediction as the estimation, being made once the task is completed, could be based on more information (i.e., on how the participants felt, its rank, the fatigue level, etc.).

A third explanatory goal was to assess if all sorts of temporal knowledge are equal. Indeed, runners probably build the task duration knowledge from the feedback they get after each training session (e.g., this session took 43 min). Thus, participants had to report what the sort of feedback they were using (1- measure of time, 2- measure of distance, 3- measure of speed), when training, to see if one sort of feedback provides a better knowledge of one’s running time that can translate into more accurate temporal prediction and estimation. We expected that using feedback about speed would be the most efficient feedback type (highest correlations with temporal precision) because one’s running speed can be applied to other (i.e., shorter or longer) running situations (e.g., if one knows s/he runs at 10 km/hr, s/he can expect to run 15 km in 90 min).

## Materials and Methods

### Participants

Ninety-one participants (50 males and 41 females) out of the 244 that were registered in a running competition enrolled in the experiment. Six participants were rejected as they did not fill the form properly or did not complete the event, leaving a total of 85 participants. The age of participants ranged from 18 to 66, with a median of 28 years old.

### Material

The participants had to fill out an in-house questionnaire assessing their sporting level, training habits and knowledge of time while running. The questionnaire was in paper form. Three questions measure training habits and were: How often do you get measures of (1) time (2) distance (3) speed when you train? The response scale extended from 1 to 5; 1 = never, 5 = always. They were also asked (on a 1–5 scale, 5 = very well) how well they know the time it takes them to run a specific distance (5 km). The other questions were “You have been participating in running race for how many years?”, “How many times per week do you run?”, “How many hours and minutes per week do you run?”, “What is your running level (amateur, provincial or national)?”, “How many times have you participated in this specific race?” “How far from your real performance would a satisfactory prediction be?”. The runners supplied their own clothing and accessories.

### Procedure

The participants first had to register for the race. The event was a local, on-campus, 5-km race open to the public, although it was also part of a provincial competitive schedule. The circuit consisted of two 2.5-km laps without any distance markers. The circuit changes every year and is unannounced, which means that runners cannot train for this specific race. The goal of the race was to finish not only in the fastest possible time, but in the most accurately predicted time (awards were also given for the best predictions). However, running as fast as possible is still the main goal of the race; the prediction process is simply added for fun. Hence, runners were not simply self-pacing to achieve a good prediction; they ran as fast as they could and hoped they predicted a precise duration. Watches or any other timing devices were prohibited. Each runner stated their predicted time when they registered for the race (and these predictions were later retrieved by the experimenters). After registration, participants were invited to enroll in the experiment. If they accepted, they had to fill out the questionnaire and return it before the start of the race. One of the questions was aimed at defining groups for statistical analyses. Thus, they had to report the level at which they compete: national, provincial, and amateur.

The race proceeded without any intervention on the part of the experimenters. They waited for the runners to pass the finish line before collecting the final running time estimates. The runners knew before the start of the race that this time estimation would be required. The runners took from 924 to 1918 s to complete the race, with a mean time of 1348 s (22 min and 28 s). It should be noted that the weather (early spring in a Northern climate) was particularly difficult with an outside temperature around 4°C wind heavy rain^1^ and gusty winds. This study was approved by the *Comité d’éthique de la recherche avec des êtres humains de l’Université Laval*, with written informed consent from all subjects. All subjects gave written informed consent in accordance with the Declaration of Helsinki.

### Data Analysis

For the purpose of comparing the effect of expertise, two groups were created: experts and intermediates. The expert group consisted of runners who compete regularly at a provincial and national level (*n* = 30). The intermediate group consisted of runners who only compete in amateur events (*n* = 55). This group allocation was based on self-reported information. Hence, in order to investigate if both groups differed in terms of running experience, the amount of training was compared. Expert runners trained in average 4.81 times a week for a total of 6.86 h per week, while these numbers are 3.14 and 2.82, respectively, for intermediate runners. The groups differ significantly for both the number of training sessions per week, *t*(83) = 5.588, *p* < 0.001, and the number of training hours per week, *t*(83) = 8.047, *p* < 0.001. Furthermore, expert runners reported they have been participating in running races for an average of 8.06 years, while this number goes down to 2.66 for novices. This difference is significant *t*(43.87) = –3.604, *p* < 0.001.

Finally, participants were asked to report how well they know the time it takes them to run a specific distance (like 5 km). Expert runners significantly reported a better knowledge (*M* = 4.14) than intermediate runners (*M* = 3.25), with scores on a 1–5 scale (5 = very high). This difference is statistically significant, *t*(78) = 5.106, *p* < 0.001. Hence, the distinction between both groups appears adequate since they significantly differ on many aspects^2^.

Two dependent variables were used for assessing performances. The first was the perceived to real time ratio (Ratio), a variable showing the side of the error (over- or underestimation). A Ratio of 1 means a perfect estimation, while Ratios under and over 1 mean time underestimation and overestimation, respectively. The second variable used was the absolute standardized error (ASE), a measure that is not sensitive to the side of the error, thus a more genuine measure of accuracy. The ASE is calculated on the ratio by taking |1-ratio|.

## Results

Table 1 shows the Ratio and ASE for the two time judgments (prediction and estimation), by expertise (experts vs. intermediates). To compare these judgments and assess if the expertise produced an effect of these judgments, a 2 × 2 factorial design ANOVA was first conducted on the Ratio, with time judgment being a repeated-measure factor and expertise a between-subject factor. The ANOVA revealed a significant expertise effect, *F*(1,69) = 7.67, *p* = 0.007, η^2^ = 0.100 and a significant interaction between time judgment and expertise, *F*(1,66) = 4.55, *p* = 0.036, η^2^ = 0.062. A breakdown of the interaction revealed that expert runners were closer to 1 than intermediate for both temporal judgments. Furthermore, for the expert runners, the estimated time was more precise than the predicted time, while there was no difference between these two temporal judgments for the intermediate runners. The same ANOVA design was used and conducted on the ASE. This time, only the effect of expertise is significant *F*(1,69) = 13.371, *p* ≤ 0.001, η^2^ = 0.109, showing that experts are more accurate for both tasks.

**TABLE 1:** **Mean (*M*) Ratio and ASE as a function of the task and expertise level**.

**Expertise level**	**Task**	**Ratio**		**ASE**	
		*M*	SD	*M*	SD
Intermediates	Prediction	1.039	0.08	0.052	0.05
	Estimation	1.027	0.05	0.043	0.04
Experts	Prediction	0.988	0.02	0.018	0.02
	Estimation	1.004	0.01	0.012	0.01

SD, standard deviation.

Since the previous analyses are based on self reported group attribution, the relation between expertise and temporal performance was further analyzed with correlational analyses. Indeed, correlations between the number of training per week and perceived time were calculated. They show that the more weekly training sessions a runner complete, the more precise the temporal judgments are, and this finding applies to both prediction (*R* = –0.575, *p* ≤ 0.001 for the ASE and *R* = –0.403, *p* ≤ 0.001 for the Ratio) and estimation (*R* = –0.498, *p* ≤ 0.001 for the ASE and *R* = –0.248, *p* = 0.036 for the Ratio).

Furthermore, runners were asked to report the frequency to which they use measures of distance, time, and speed. Correlational analyses were conducted to assess if the use of a specific feedback was associated with temporal accuracy (again using the percentage of error). The analyses revealed that the use of speed was the only feedback type that correlated significantly with time prediction (*R* = –0.285, *p* = 0.019 for the ASE and *R* = –0.239, *p* = 0.033). Thus, the more runners reported using measures of running speed while training (regardless of their expertise levels), the more precise was their predicted time. A mediation analysis revealed that the use of speed-related feedbacks did not mediate the effect of expertise. Although correlated to predicted time, the usage of feedback was not correlated to estimated time.

## Discussion

This section will first discuss about the effect of extensive training on temporal performance and secondly, will contrast the prediction and the subsequent estimation.

### Effect of Experience

The results show that expert runners are better at predicting their running time than intermediate runners. This conclusion is coherent with other studies showing prior task experience enhances the prediction accuracy (Boltz et al., 1998). While there is sufficient body of studies showing this role of prior task experience on temporal prediction, the demonstrations were usually made with very limited prior task experience or training with the task. Hence, the participation of experienced runners allowed assessing how extensive training affects the accuracy of the prediction.

Both groups of runners exhibited surprisingly unbiased predictions. Indeed, compared to other studies using temporal prediction (see 1 in Roy et al., 2005), the ratios recorded here are quite close to 1. Hence, it suggests that the more one is experienced with a task, the better the prediction becomes. That is coherent with Tobin and Grondin’s (2012) study in which experimented athletes reached an accuracy level on a temporal estimation task much better than what is generally observed in the literature for similar tasks/durations. Consequently, both studies converge and show that temporal perception processes (estimation or prediction) are strongly affected by prior task experience and that a “near-perfect” ratio is possible with sufficient training with the temporal task.

Another aspect of the results is interesting. Indeed, not only were the expert runners more accurate, the side of their error (over- or underestimation) was the opposite than the one observed with intermediate runners. Indeed, expert runners predicted a faster performance than what they actually accomplished while intermediate runners underestimated their performance by predicting a slower time. The amount of prior task experience not only affected temporal precision, but also caused a directional effect. This directional effect may be caused by one’s confidence into personal abilities, with experts being more confident than intermediates.

While these results show that experts are better at perceiving time, little is known as to *why* exactly they are better. Tobin et al. (2010) studied the time perception of gamers for 12 and 35 min of gaming. In their studies, gamers reported playing an amount of 12.95 h per week on average. This amount of game play exceeds by far the amount of training reported here by the runners. However, gamers were quite imprecise at estimating time, with ratios ranging from 1.2 to 1.6 depending on the duration used. Thus, it appears that doing a specific task often, be it running or playing video games, is not sufficient to create temporal expertise. The main difference between these two tasks can be the importance of time. Indeed, when playing, the duration of the game is not important. In fact, many players reported they specifically play to lose track of time (Wood et al., 2007). However, when training, runners may pay close attention to their distance, time and speed. Hence, for the large experience with the task to translate into more accurate temporal perception, it might be necessary to pay attention to the duration of each activity (i.e., each training session) and get timely feedback (e.g., this 5-km training took 21 min). Without these feedbacks, temporal expertise may not develop, like in the case of gamers. Indeed, it is well known that temporal feedback improves time perception (Fraisse, 1971; Hicks and Miller, 1976; Ryan and Fritz, 2007).

This explication is also coherent with the memory bias account proposed by Roy et al. (2005). Indeed, they suggested that the inaccuracy in the temporal prediction could be caused by an inaccuracy in the memory of the previous occurrence of the task. Said differently, people have poor prediction because they remembered poorly the duration it took the previous times. Thus, receiving timely feedback may often help creating an accurate memory of how long the task last, which in turn translates into accurate predictions. In line with this idea, runners had to report what kind of feedbacks, if any, they use while training (elapsed duration, traveled distance, or averaged speed) to see if the use of these feedbacks correlates with temporal performance.

The results show that, among time, speed and distance, it is only the usage of speed-related feedback that is significantly correlated with the accuracy of time prediction, regardless of the expertise level. Hence, the more a runner uses a measure of speed when training, the more precise at predicting time he/she becomes. This finding suggests that runners could gain their temporal expertise through the feedback they got after each training session (in fact, many GPS systems nowadays seem to have this in mind, helping runners know their running pace when training). Indeed, by learning their average speed, it becomes easier for them to know how long running a specific distance should take by using a simple formula based on their average speed.

### Prediction vs. Estimation

The second main goal of the experiment was to contrast the initial temporal prediction with the estimation upon completion. As stated in the introduction, few studies compared the performance level on a temporal prediction with its subsequent temporal estimation. Furthermore, the conclusions from such studies differed, offering quite a complex picture. Based on our results, both the accuracy of the initial prediction and the expertise level of the participant might explain the difference between prediction and estimation and further explain why different studies reached different conclusions.

First, for novel or occasional tasks such as the one used by Burt and Kemp (1994), the recorded predictions were far from accurate. Hence, once the task is completed, participants may easily figure that their prediction was wrong and adjust it with a more precise estimation. This could explain why in such cases the estimation is more accurate than the prediction. Indeed, the farther the prediction is from the actual duration, the larger are the chances to improve the subsequent temporal estimation as there is much more room for improvement.

However, when the prediction accuracy is closer to the target duration, it may take a certain level of expertise to be able to adjust that prediction and make a more precise estimation. Indeed, our intermediate runners did not improve their prediction accuracy when estimating time after completion. Similarly, novice pianists in the Boltz et al.’s (1998) experiment only improved in the 2° of familiarly (novel and well trained) for which their predicted time was the less accurate (however, for the extremely well trained melody, the prediction of novices was more accurate and their estimation did not improve that prediction). On the opposite, our expert runners and Boltz et al.’s (1998) expert pianists were always better at estimating than predicting time, even if they were better than novices at predicting time. Hence, it may require a certain level of expertise with the task in order to “read” the duration of the task and correct the prediction into a more precise temporal estimation. Thus not only prior task experience or expertise would predict the accuracy of the prediction, but it would also predict one’s ability to make a temporal estimation that is more accurate than its initial prediction.

### Limitations and Future Studies

Relying on athletes allowed testing an amount of training that is almost impossible to recreate in a laboratory setting. As the insufficient amount of training in other studies to fully reflect “real-life” situations was an important issue, the participation of athletes was a sound choice. However, the clear drawback from this decision is that participant came to the study with their own background; it was thus impossible to monitor their training. Since we advocate for more ecological studies in timing (see Tobin et al., 2010), especially when studying prior task experience, we argue that this limitation is minor. However, subsequent studies with more experimental control on the training process will be necessary to better understand how prior experience improves timing. Especially, monitoring the training process could be very informative and might show the learning curve (for instance, what amount of training is required to reach an asymptotic temporal performance?).

It could be argued that another limitation of the present study is the fact that the groups were separated on the basis of self-reported data (expert or intermediate). However, the statistical analysis made on the amount of training actually showed both groups do differ significantly. Furthermore, correlation analyses showed that the more runners train, the more accurate their temporal perception is. This key finding is independent from the group attribution.

## Conclusion

This study adds to the large body of evidence showing that prior task experience enhances temporal prediction accuracy. Furthermore, the participation of athletes showed that with more experience with a task, predictions get more accurate. It further shows that extensive training improves temporal performance up to an impressive level. This finding also applies to the temporal estimation made after the task’s completion. Finally, the difference between the prediction and the estimation of a task may depend on both the accuracy of the prediction, and the level of experience with a task.

### Conflict of Interest Statement

The authors declare that the research was conducted in the absence of any commercial or financial relationships that could be construed as a potential conflict of interest.

## Footnotes

1 According to verbal reports of many participants, the climate conditions slowed the overall running performance. However, they did report taking the weather into account when registering the temporal prediction.

2 The experts reported here may not represent “real” experts by some as they are not elite international level. However, the significant differences between the two groups reported here are strong enough to represent two distinct groups having a distinct background with running. It is not a study aimed at extraordinary elite experts.
